# Fœtal Skeleton Passed per Rectum

**Published:** 1891-12

**Authors:** W. P. Powell

**Affiliations:** Willis, Texas


### Correspondence.

### FŒTAL SKELETON PASSED PER RECTUM.

WILLIS, TEXAS, Nov. 6th, 1891.

*Editor Daniel's Texas Medical Journal:*

Allow me the privilege of reporting another one of those rare, interesting cases,—the first and only one occurring in my practice in this section of the State for nearly thirty-three years—with correct diagnosis and prognosis.

On November 6th, 1890, I was called to see a colored woman, the wife of Wm. Collins, near Willis, who had been in bed two weeks or more. She had pains resembling labor pains, and threatening abortion. She had the appearance of six or seven months pregnancy. Principal portion of tumor occupying the left hypogastric region and above, but extended over whole of abdomen. Vaginal examination revealed the tumor low down, exceedingly hard to the touch; no discharge per vaginum, but a whitish mucus passing from bowels upon evacuation of same. The os could not be detected, it being above the lower edge of tumor, the pressure of which was so firm against the vaginal walls, so thoroughly filling the pelvic cavity as to make it impossible to reach the os. Upon examination of abdomen I could detect a circumscribed elongated tumor, from the vaginal portion to its upper abdominal portion, to be eight or nine inches—the upper end above umbilicus—enlargement passing over and below umbilicus to near crest illium. Very firm on left side near top of tumor, I could detect, as I thought, a soft sack, containing fluid of some kind, about the size of a goose egg. This I took to be the sack of waters.

My diagnosis of this case was extra uterine fœtation.

I told the parties interested that it was my conviction, and the only safe and speedy relief, laparotomy, which was most positively rejected by the woman—rather risk death as she was. I then remarked to them, that sometimes nature made desperate efforts to relieve itself; possibly, in time, the skeleton may pass through a fistulous opening in the colon made by ulceration, and pass out at the rectum. I also told them if the fœtus should continue to grow and they refuse again laparotomy, I would do all in my power for her—try the use of electricity to destroy life of fœtus. However, during this interval I would do what I could to sustain the system and create absorption until the crisis came. For this, my prescription ran thus:

| | | |
|---|---|---|
| ℞ | Iod. Pottassium | ℥jss |
| | Bichloride Mercury | grs. ij |
| | Fluid Extract Stillingia | ℥ij |
| | Fluid Extract Sarsaparilla | ℥ij |
| | Water | qs. ℥ viij |

M. Sig. 1 teaspoonful after each meal.

This prescription was filled only twice. Sulphate morphine was given only as required to keep down pains, and to be kept perfectly quiet.

This was my last visit to patient for nearly twelve months. In two months patient walked to my office, two miles, had very little pain occasionally; tumor about same size, but she thought at times softer, but a little tenderer upon pressure. In about one month from that time, patient visited my office again. Tumor somewhat reduced, and continued to reduce or contract. General health improving rapidly; pains disappeared entirely; perfectly well except hard lump which receded to two or three inches below the umbilicus, contracted in its general and long diameter.

This patient engaged in farm work, and assisted in making and gathering a crop of corn and cotton without any inconvenience. Toward the close of the farm season she visited relatives in a neighboring village, spent a week or more, returned home and was taken sick with slight fevers for a week or more, when I was called to see her, nearly twelve months from the last visit; found her with fever of a low grade, without abdominal pains; the abdomen assuming nearly its normal proportions; the tumor occupying the pelvic cavity, and apparently the exact position of the uterus, and felt hard, firm and nodulæ, but little tender, resembling a carcinomatous womb; the womb not distinguishable from tumor. I advised her to go back to her tonic and alterative treatment and morphine in case of pain.

About one week from this visit another M. D. was called in. He pronounced it a tumor; did not learn what kind.

About one week after this, the husband reported to me that some dysenteric white mucus was passing, when she had action, from the rectum. Seeing such an anatomical change, from six or seven months abdomen to a tumor not larger than two oval hands laid together, I began to wonder, in the absence of other evidence, if I had made a proper diagnosis twelve months ago. Ordered C. to keep the bowels well washed out with hot water; possibly I might be mistaken, and it rusult in cancer; to sustain the strength with diet and tonics until time would develop this strange phenomenon.

In a week after this, I was called to see a patient in an adjoining county. C. came for me in haste. Not willing to wait, he called in a physiciam most convenient. His attention called to the situation, he examined the rectum and found the fœtal skeleton passing through that channel. He thinks he has it entire. She is now doing well. If the bones of the head have been removed, I cannot see why she will not make a good recovery.

Thus you see, once again, the great surgical powers of nature.

I have given all symptoms and anatomical changes that would be of interest to the medical profession.

Rare as it is, and hopeless as it seemed to me at the beginning by refusing laparotomy, nature says by her action in this case that our modern surgery is not yet complete, and without her none are saved.

I understand the doctor who delivered the fœtal skeleton reported it to the *Medical Brief* with unbecoming reflections.

If you think proper, comment on this very interesting case to me.

Respectfully,

W. P. POWELL.

